# Which preventive control measure initiated the “flattening of the curve”: A comparison of Austria and Victoria during the 2nd wave of the COVID-19 pandemic

**DOI:** 10.1007/s00508-022-02108-w

**Published:** 2022-11-24

**Authors:** Franz Konstantin Fuss, Yehuda Weizman, Adin Ming Tan

**Affiliations:** 1 Chair of Biomechanics, Faculty of Engineering Science, University of Bayreuth, Universitätsstraße 9, 95440 Bayreuth, Germany; 2 Faculty of Health, Arts and Design, Swinburne University, VIC 3000 Melbourne, Australia

**Keywords:** COVID-19, Lockdown, Effectiveness, Reproduction number, Policymaking

## Abstract

**Background:**

When a country introduces different COVID-19 control measures over time, it is important to identify the specific measure that was effective and therefore responsible for “flattening the curve”. This information helps policymakers find the right decision and saves the economy by avoiding severe yet ineffective measures.

**Objective:**

This paper aims to fill the literature gap by investigating two regions that introduced two or three consecutive measures during the second COVID-19 wave, namely Austria and Victoria.

**Method:**

We calculated the first derivative (acceleration) of the filtered daily case data and identified the date of the start and end of the acceleration’s major downturn (effective phase) relative to the date when the control measures were introduced (Austria: soft/hard lockdowns; Victoria: stages 3/4 lockdowns, mask order).

**Results:**

In Austria, the effective phase started 5 days after the introduction of the soft lockdown and ended at the start of the hard lockdown. In Victoria, the effective phase started 19 days after the introduction of the stage 3 lockdown, 5 days after the introduction of the mask order, and ended 6 days after the start of the stage 4 lockdown.

**Conclusion:**

Considering that the effect of control measures is expected the earliest one serial interval after their introduction, the control measure responsible for “flattening the curve” was the soft lockdown in Austria and the mask mandate in Victoria. The severe lockdowns in both regions were ineffective.

## Introduction

“Flattening the curve” is a public health strategy intended to slow down the spread of a virus, specifically applied during the coronavirus disease 2019 (COVID-19) pandemic. The term “curve” in this context usually refers to the cumulative case numbers. The preventive control measures introduced as strategies for “flattening the curve” during the first country-specific COVID-19 wave covered a wide range [1, 2], from light to forced measures, such as: social distancing in public, wearing face masks in public, prohibition of outdoor and indoor gatherings, closure of educational facilities, closure of non-essential businesses, stay home and work from home policies, movement restrictions, quarantine measures, contact tracing, permits for travelling to work, night time curfews and combinations thereof usually referred to as “lockdowns”, with measures enforced by law and penalties. Different control measures can be introduced simultaneously, or incrementally with increasing severity. In the latter case, it is essential to understand which measure was responsible (in combination with preceding measures) for “flattening the curve”.

**Fig. 1:**
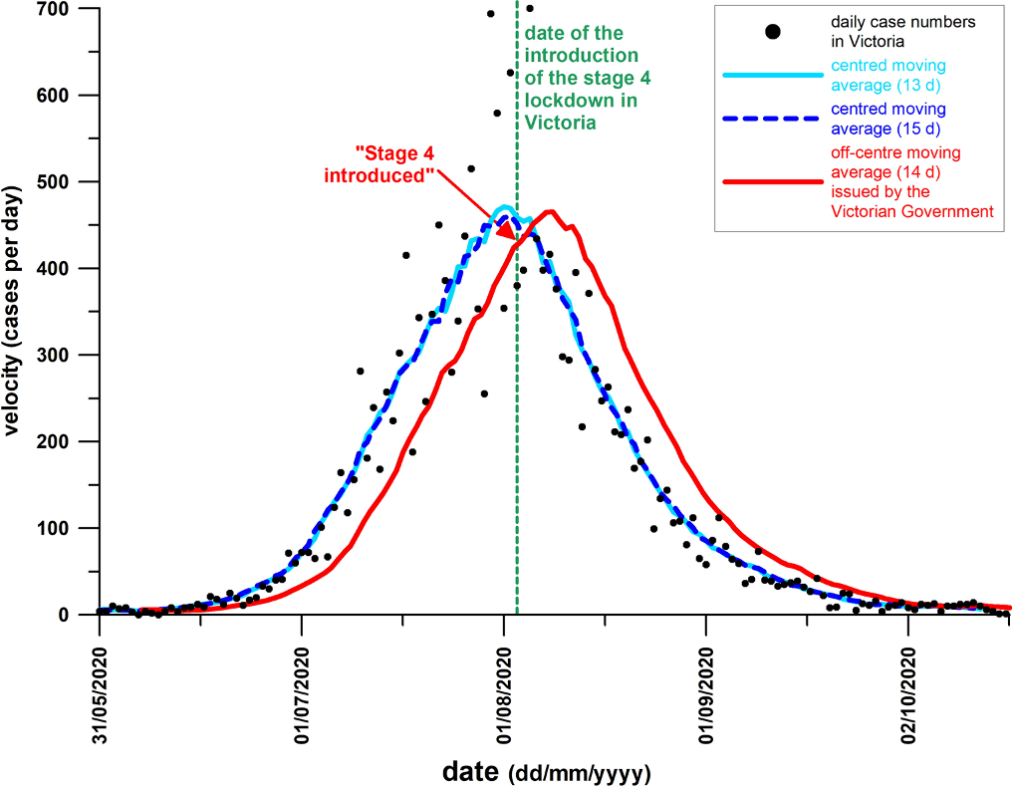
Victorian daily case numbers and comparison of centred and asymmetric moving averages; the off-centre, asymmetric average (window width of 14 data, phase shift 6.5 days) was used by the Victorian Government (as shown by ABC News [4]), which is the same as MS Excel would calculate the moving average; while the stage 4 lockdown could have caused and initiated the effective phase based on the asymmetric moving average, it happened 1 day after the peak average based on the centred moving average and therefore was too late for being causally linked to the stage 4 lockdown (cf. Fig. 5)

How can we identify a point on the cumulative case curve, that tells us where the flattening starts? The first time-derivative of the cumulative case numbers is worth exploring. The new daily case numbers, i.e., the velocity of the disease spreading [3] are, strictly speaking, not exactly the first derivative of the cumulative case numbers as the cumulative case numbers are the sum of the daily increase in cases but not the integral of the daily case numbers. Nevertheless, the daily case numbers show a distinct marker (in contrast to the cumulative case numbers) at their peak datum. In fact, the rolling average of the daily case numbers was used by the Victorian Government ([4]; Fig. 1, red curve) to exemplify the successful effect of the stage 4 lockdown. Fig. 1 (red curve) shows that the average daily case numbers reach their peak only 6 days after introducing the stage 4 lockdown, which, according to the Victorian Government, causally links the downturn of the daily case numbers to this specific control measure: “less stringent stage 3 restrictions have proven ineffective in Victoria” [4]; “stage 4 restrictions enabled Victoria to turbo-charge its exit from wave 2” [4].

Saul et al. [5] used the gradient of the natural logarithm of the daily case data (i.e., the logarithmic growth rate [6]) to verify whether the stage 3 lockdown of Victoria (starting 9 July) “reduced the transmission of COVID-19”. Fuss et al. [3] used the gradient of the daily case data to define the start and end of the effective phases of control measures (irrespective of their nature) and calculated their effectiveness from the 2nd time derivative of the daily case data; however, Fuss et al. [3] evaluated the difference between severe and relaxed control measures and compared these measures across 92 countries, but not within the same country. The same principle applied to Haug et al. [7] who compared between countries, but not within the same country. They identified the most successful control measures from evaluating and ranking the effectiveness based on the change of the effective reproduction number.

The aim of this study is to identify the control measure responsible for “flattening the curve” if a specific country introduces different measures over time. The methodological approach is exemplified by two regions that used a similar strategy of introducing two different kinds of lockdown at different times. The applicability of our method is supported by further data from 18 countries that introduced a series of consecutive control measures.

## Methods

### Choice and rationale of method

**Fig. 2:**
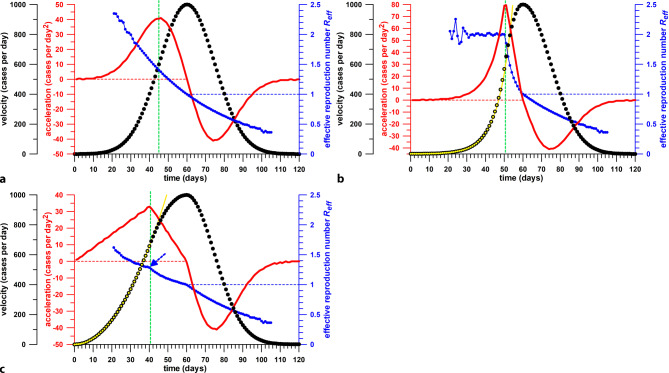
Three scenarios of daily case numbers (**a** Gaussian, **b** exponential, **c** quadratic), their 1st time derivative (acceleration), and effective reproduction number R_eff_; the *vertical green line* marks the beginning of the effect introduced by control measures

Instead of referring to the peak case data as shown in Fig. 1, the effective reproductive number *R*_*eff*_ could have been consulted, at the transition from epidemic to endemic, where *R*_*eff*_ = 1; however, as Fuss et al. [3] pointed out, the start and the end of the “effective phase” of control measures cannot be determined from *R*_*eff*_, but rather conveniently and exactly from the acceleration of the spreading viral disease, i.e., the time derivative of the velocity data (daily case numbers); however, the acceleration and *R*_*eff*_ are inherently related mathematically, as both are calculated from the gradient of the new daily case numbers, the acceleration from the original velocity data, and *R*_*eff*_ from the natural logarithm of the velocity data. The gradient of the natural logarithm of the velocity data corresponds to the logarithmic growth rate *K*. When using the exponential method of Diekmann et al. [6], *R*_*eff*_ is calculated from e^*K SI*^, where *SI* is the serial interval. Calculating the time derivative from the natural logarithm of the velocity data obscures the acceleration peaks, otherwise clearly visible from the derivative of the original velocity data. This behaviour is exemplified in Fig. 2. In an exponential concave-up growth profile (Fig. 2a), at the transition to a concave-down polynomial, the acceleration shows a distinct peak, and the initially constant *R*_*eff*_ exhibits a sharp drop. In a sub-exponential concave-up growth profile (quadratic growth; Fig. 2b), the acceleration peaks again at the transition, whereas the *R*_*eff*_ is monotonically decreasing, with a minute cusp at the transition. In a Gaussian concave-up growth profile (Fig. 2c), there is no distinct *R*_*eff*_ marker coincident with the acceleration peak. In real-world data, cusps can appear or disappear due to noise and filtering thereof, respectively.

**Fig. 3:**
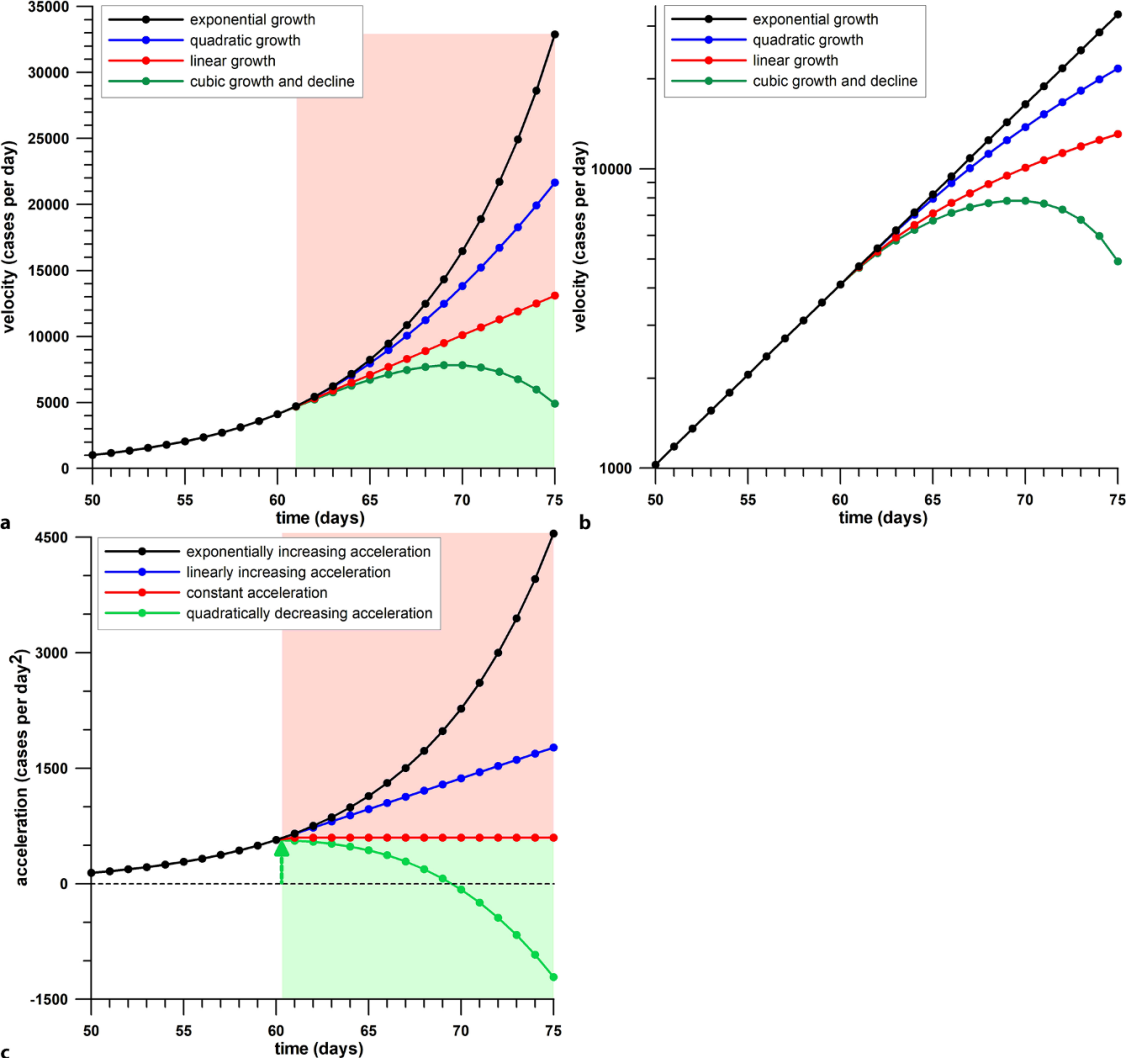
Scenarios of daily case data (velocity) and their time derivative (acceleration) against the time; **a** functions other than exponential, start on day 61. (*quadratic growth* subexponential growth, *light green area* effect to be achieved by control measures, *pink area* missing or ineffective control measures), note that there is only one concave-down function (cubic) in subfigure (**a**); a concave-down function is required for initiating the effective phase; **b** logarithmic data representation pretends to have three concave-down function (quadratic, linear and cubic); **c** only the decreasing acceleration has a peak point on day 60 (*green arrow*, indicating the beginning of effective phase, and leading to flattening the curve); the constant acceleration is a borderline case, separating the positive and negative gradients; the exponential and linear accelerations have not reached the effective phase yet

Fuss et al. [3] are referring to a “force” generated by control measures, that is required for interrupting the natural growth (daily case data; velocity) and for bending the slope of the velocity curve from concave-up to concave-down, such that the acceleration decreases. This scenario is shown in Fig. 3. In an exponentially growing velocity curve (Fig. 3a), the acceleration (Fig. 3c) increases exponentially. Bending the curve such that the initially exponential velocity curve becomes sub-exponential (e.g., quadratic) at a later stage is ineffective, as the acceleration is still increasing (linearly in this case). Bending the velocity curve to the extent that it becomes linear results in a constant acceleration, which neither increases nor decreases, with pending effectiveness. Only if the acceleration drops, is a distinct peak visible in the acceleration profile (Fig. 3c), by a sufficient amount of force related to control measures. This acceleration peak is the start of the effective phase [3] of a preventive control measure. If there was any measure introduced shortly before the acceleration peak, then there is a causal relationship between this preventive measure and the downturn of the acceleration curve. The term “shortly before” requires definition. Control measures are supposed to interrupt the transmission of the virus between people, and the further infection of sound people; however, people infected before the introduction of the control measure during a time period smaller than *SI*, will be detected as new cases only after the introduction date over a time period smaller than *SI*. The daily case data will therefore continue to grow uninterruptedly for approximately *SI*. In this context, the term “*SI*” serves only as an approximation, as the actual time period of uninterrupted growth depends not only on (1) how the new cases are detected (tests and/or symptoms), on (2) the actual durations of latent period, infectious phase, incubation phase, and the average time from infection to test result but also on (3) compliance with, and enforcement of, control measures and on (4) filtering of the naturally noisy daily case data.

The average (or median) of the serial interval (*SI*, in days) varies between different sources: 3.95 (Tianjin [8]), 3.96 [9], 4.0 [10], 4.46 [11], 4.6 [12], 5.2 (Singapore [8]), and 7.5 [13]. Furthermore, *SI* follows a gamma distribution with a probability density of 0.097, 0.21 (peak) and 0.015 for *SI* = 1, 3, and 10 days respectively [11]. In addition to the *SI*, the effect of a control measure may be delayed by low compliance. We therefore suggest that the onset of the control measure’s effect happens between 5 and 10 days after introducing the control measure.

### Countries/states investigated

The daily new case data (velocity) of Austria and Victoria were obtained from publicly available data [14, 15]. Both Austria and Victoria introduced 2 different lockdowns to battle the 2nd COVID-19 wave:

Austria:

- 3 November 2020: soft lockdown [16]: curfew (2200–0600, with exceptions); social distancing (min. 1 m); wearing face masks in public, indoors and on public transport; working from home is recommended; closure of restaurants, leisure and recreational facilities, upper secondary schools and universities; prohibition of public and private events including contact sport.
- 17 November 2020 (14 days after soft lockdown): Hard Lockdown [17]: on top of the Soft Lockdown rules: total restriction of movement (leaving home only for the following reasons: work, medical care, shopping of essential goods, physical and mental recreation, caregiving); no contact with people living outside the household (except partners and a single relative); closure of non-essential businesses, primary and lower secondary schools, and all sports facilities.

Victoria:

- 9 July 2020: Stage 3 Lockdown [18]: social distancing (min. 1.5 m); closure of pubs, bars, entertainment venues, churches and places of worship; restricting restaurants and cafes to take-away only; limiting public gatherings to 2 people; movement restriction to maximally 20 km from home; working from home (with exceptions); stay at home except for four reasons: shopping for food and supplies, providing care and caregiving, exercising, and studying and working if it cannot be done from home.
- 23 July 2020 (14 days after stage 3 lockdown): mandatory mask order [19] in public spaces indoors and outdoors (this was the first time that wearing face masks was mandatory in Australia; Austria introduced masks already during the 1st wave, on 6 April 2020 in stores, and on 14 April on public transport).
- 3 August 2020 (25 days after stage 3 lockdown): Stage 4 Lockdown [20]: on top of the Stage 3 Lockdown and mask order rules: curfew (2000–0600, with exceptions); no change of sleeping place (with exceptions); movement restriction to maximally 5 km from home; prohibition of weddings; exercise limited to 1 h max and 2 people max; shopping limited to 1 person per household per day (with common sense exceptions); closure of primary, secondary and tertiary education facilities; closure of non-essential business; permits required for working outside home.

Additional countries/states:

We analysed data of an additional 18 countries and states that introduced control measures during the 1st COVID-19 wave. This investigation served to apply the method suggested above to identify the effect of consecutive control measures, as well as the timeliness of single control measures, i.e., whether they were introduced too early or too late and thus did not have the desired effect. The control measures introduced were lockdowns as per the definition of Fuss et al. [3] in addition to mask mandates in 17 countries. In Germany, there were three consecutive restrictions such as cancellation of large public events (9 March 2020); closure of educational facilities (schools, childcare) and many stores (16 March 2020); and a general contact ban (23 March 2020), prohibiting small public gatherings and closing restaurants and non-essential retail [21].

### Data processing and analysis

**Fig. 4:**
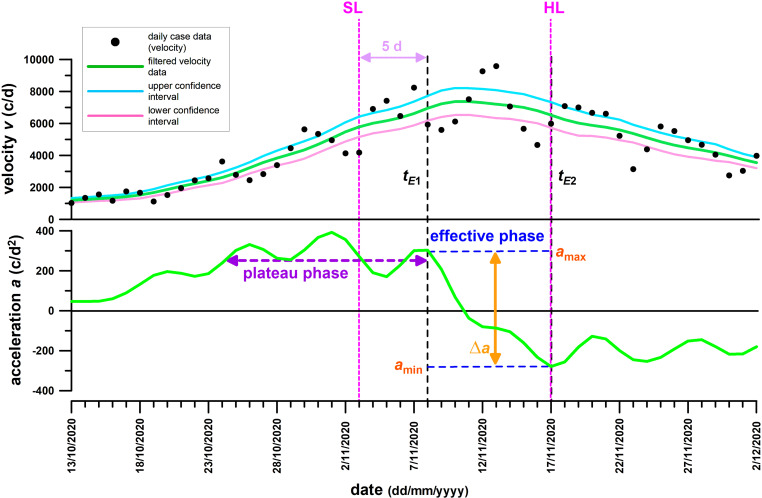
Austria’s velocity (daily cases) and acceleration data. *SL* date of soft lockdown, *HL* date of hard lockdown, *c* cases, *d* days, *t*_*E1*_* and t*_*E2*_ start and end of the effective phase, *a*_*max*_* and a*_*min*_ acceleration peaks at t_E1_ and t_E2,_ *∆a* decrease of the acceleration during the effective phase

**Fig. 5:**
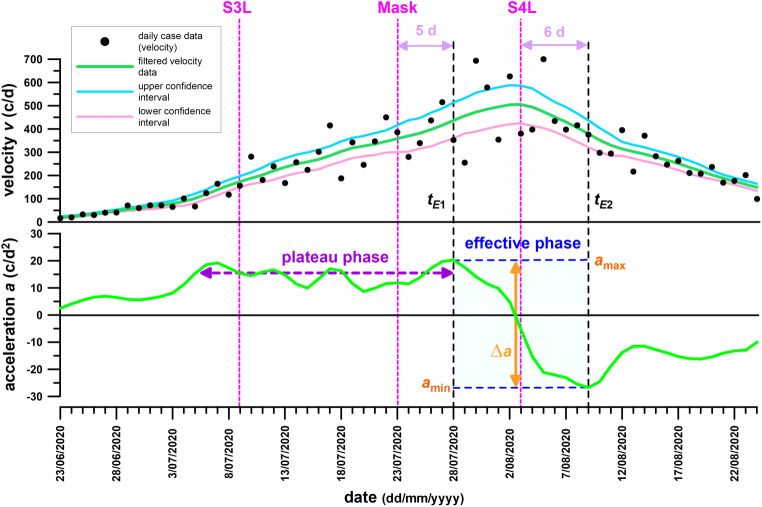
Victoria’s velocity (daily cases) and acceleration data. *S3L* date of stage 3 lockdown, *Mask* date of the compulsory mask order, *S4L* date of stage 4 lockdown, *c* cases, *d* days, *t*_*E1*_* and t*_*E2*_ start and end of the effective phase, *a*_*max*_* and a*_*min*_ acceleration peaks at t_E1_ and t_E2,_ *∆a* decrease of the acceleration during the effective phase

The daily new case data (velocity) were filtered with the method of Fuss et al. [3] by using a double symmetric running average filter with a window width of 3 data, followed by a symmetric running quadratic filter (2nd order Savitzky-Golay filter [22]) over a window of 13 data. The filtered velocity data were numerically differentiated to obtain the acceleration. The effective phase of control measures was identified from the acceleration crossing the zero line, between the last highest preceding positive peak, and the following first highest negative peak [3], as shown in Figs. 4 and 5.

## Results

The daily case data (velocity), raw and filtered, as well as their acceleration, of Austria and Victoria are shown in Figs. 4 and 5.

### Austria

The acceleration of daily case data (velocity) increased from zero to a point, approximately 10 days before the start of the soft lockdown, where the acceleration remained constant on average with local fluctuations (Fig. 4). This plateau phase ended 5 days after the start of the soft lockdown, with a final peak before the acceleration rapidly decreased, crossed the zero line, and became negative, and finally reached a maximum negative peak 9 days after the last positive peak. The start of the effective phase 5 days after the introduction of the soft lockdown suggests that the soft lockdown was responsible for initiating the effective phase and that the soft lockdown was effective over 9 days, after which it reached its natural capacity. Precisely at the end of the effective phase, the hard lockdown started, which was unable to extend the effective phase by decreasing the acceleration further, and which was therefore ineffective.

### Victoria

Comparable to the Austrian data, the acceleration increased first and then reached a plateau phase approximately 4 days before the start of the stage 3 lockdown (Fig. 5). This lockdown was unable to initiate the effective phase so that the acceleration remained constant on average with local fluctuations. This plateau phase ended 5 days after the introduction of the mandatory mask order, which was therefore responsible (on top of the ineffective stage 3 lockdown) for initiating the effective phase. The latter lasted for 12 days and ended 6 days after the introduction of the stage 4 lockdown. Even 2 days after this lockdown, the acceleration curve flattened and finally reached a maximum negative peak. The stage 4 lockdown was not able to extend the effective phase further, and was therefore ineffective, in the same way as the hard lockdown was in Austria.

### Further countries/states

**Fig. 6:**
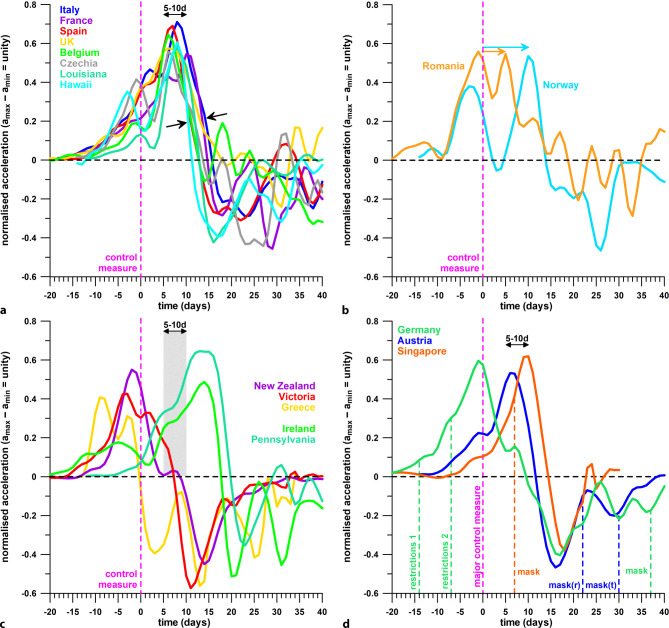
Normalised acceleration data versus time, in relation to the time of introduction of control measures (time = 0 in case of lockdowns, or the 3rd restriction in Germany); **a** countries/states with the beginning of the effective phase 5–10 days after the introduction of the control measure (the 2 *arrows* indicate the major downturn of the acceleration, resulting in deceleration of the daily case data); **b** countries with 2 acceleration peaks (the 2 *arrows* indicate the peaks after the introduction of the control measure); **c** countries/states with the beginning of the effective phase before or more than 10 days after the introduction of the control measure; **d** countries with 2–3 consecutive control measures

Fig. 6a shows eight countries/states whose lockdown was introduced 5–10 days before the beginning of the effective phase. The data suggest that these lockdowns were successful.

Fig. 6b shows 2 countries with 2 acceleration peaks, the 1st one occurring 3 and 1 days before the date of the lockdown, and the 2nd one, 10 and 5 days afterwards (Norway and Romania, respectively). The beginning of the effective phase was clearly at the 2nd peaks, followed by the major downturn of the acceleration curves. Both lockdowns were successful.

Fig. 6c shows three countries/states with the beginning of the effective phase before the introduction of the lockdown, suggesting that lockdown came too late. In 2 countries/states, the effective phase started on day 14 after the lockdown, as the effect of the lockdown was severely delayed.

Fig. 6d shows three countries with consecutive restrictions.

In Germany, the first 2 restrictions were introduced 13 and 6 days before the beginning of the effective phase and the 3rd, the major control measure, 1 day after the beginning of the effective phase. The data suggest that the 2nd restriction, in combination with the 1st one initiated the flattening of the daily case data (growth rate), whereas the 3rd one came too late and did not contribute to this initiation. It may have contributed to further decelerating the daily case data.

In Austria, the lockdown was introduced 6 days before the beginning of the effective phase, whereas the 2 mask mandates happened after the end of the effective phase.

In Singapore, the lockdown was introduced 10 days, and the mask mandate 3 days, before the beginning of the effective phase. It is therefore likely that the lockdown was solely responsible for flattening the daily case data, whereas the effect of the mask mandate followed only some days later.

## Discussion

Figures 5 and 1 (blue curves) stand in contrast to Fig. 1 (red curve). While Figs. 5 and 1 (blue curves) identify the introduction of the stage 4 lockdown slightly after the peak of the daily case data, Fig. 1 (red curve, comparable to diagram issued by the Victorian Government [4]) shows this peak 6 days before introducing the stage 4 lockdown. The reason for this discrepancy is due to a phase shift, as shown in Fig. 1.

According to the definition of the Australian Bureau of Statistics [23]:

“A phase shift is the time shift between the filtered cycle and the unfiltered cycle. A positive phase shift means that the filtered cycle is shifted backwards and a negative phase shift it is shifted forwards in time.”

“Phase shifting occurs … for example when the moving average is placed off-centre by the asymmetric filters. … Odd length symmetric moving averages (as used by the ABS), where the result is centrally placed, do not cause time phase shifting.”

The reference to the ABS is used here on purpose, as the ABS is an agency of the Australian Government, and it is assumed that the Victorian Government uses the guidelines of the ABS. Odd length symmetric moving averages are the standard method when calculating moving averages without a software package. Microsoft Excel’s built-in Trendline Option of a Moving Average, for example, has a negative phase shift of (*n* − 1)/2 data points, where *n* is the amount of data of the running window (which was 14 in Fig. 1, red curve). Evidently, the maximum of skewed daily case data is not identical to the maximum of its symmetric sliding average; however, as seen from Fig. 1, the daily case data are quite symmetrical and the blue curves are well placed inside the scattered dataset, whereas the red curve, applied by the Victorian Government, is shifted to the right, with its descending shank being entirely outside the dataset.

Undeniably, Governments must bear a heavy responsibility towards taking the right steps, for minimising the damage done by control measures. A lockdown too light might not flatten the curve but save the economy, whereas a lockdown too hard has the opposite effect and in addition affects the compliance of the population. To save the face of Governments, it must be emphasised that the appropriateness of a decision cannot be seen immediately but only retrospectively. For example, to calculate a specific datum from a moving window filter (be it a linear, i.e. average, or a higher order Savitzky-Golay [22] filter) on day *x*, the raw data until day *x* + (*n* − 1)/2 are required, where *n* is the window width. If the criterion for the success of a control measure is the acceleration reaching zero, with the effect becoming visible at the last acceleration peak before the major downturn towards zero, then the time from last peak to zero acceleration adds to the time delay. And so does the period between introduction of the control measure and the onset of its effect, which was explained from the *SI* and other factors above. If we take the time interval from the effective control measure (retrospectively seen) to zero acceleration, plus (*n* − 1)/2, then the effect becomes apparent with a delay of 12 days for Austria, and 11 days for Victoria. Adding a couple of days to ensure that the acceleration stays negative is required for being on the safe side.

Saul et al. [5] used the gradient of the natural logarithm of the daily case data to confirm whether the stage 3 lockdown of Victoria (starting 9 July) “reduced the transmission of COVID-19”. They quantified this reduction in terms of the effective reproduction number *R*_*eff*_. We already addressed the problems in the *Introduction*, when using *R*_*eff*_ for predicting which control measure caused the acceleration becoming negative. The main issue is, when using a Gaussian daily case (velocity) profile, that after log-transformation of the daily case data, the daily case function becomes quadratic and the thus the gradient is linearly decreasing. This means that the first time-derivative of the log-transformed daily case data is decreasing anyway. Nevertheless, the sudden change in the gradient of the log-transformed daily case data detected on 7 July by Saul et al. [5] corresponds to the first local acceleration peak of the plateau phase (Fig. 5); however, we cannot deduce that the associated control measure (stage 3 lockdown in this case) is effective, when using the gradient of the log-transformed daily case data. In fact, it is ineffective (Fig. 5). The reason for this is seen in Fig. 3. When log-transforming the daily case data of Fig. 3a (as shown in Fig. 3b), then the 4 different curves of Fig. 3a change their shape: concave-up exponential becomes straight in Fig. 3b; concave-up quadratic (sub-exponential) becomes concave-down; straight linear growth (constant acceleration) becomes concave down; and concave-down cubic remains concave-down. As a concave-down curve is the aspired goal of control measures, only concave-down cubic is the preferred option in Fig. 3a. In Fig. 3b, however, even the concave-up quadratic (sub-exponential) function of Fig. 3a becomes concave-down in Fig. 3b, and thus seems to be optimal, which is a deceptive and misleading conclusion, as the acceleration of the daily case data is still increasing.

Saul et al. [5] “assumed that the introduction of compulsory masks in Melbourne at midnight on 22 July would not have affected the daily cases over the subsequent 7 days to 30 July” and therefore they defined the postintervention period (i.e., after the stage 3 lockdown) from 10 to 30 July 2020. This assumption turned out to be incorrect. Our results showed clearly that the compulsory mask order was the trigger (on top of the stage 3 lockdown) that reduced the acceleration of the daily case data. This effect is even more important as the contribution of masks to interrupting the transmission of COVID-19 was underestimated, if not grossly neglected. Austria imposed the mask order only on 6 and 14 April 2020 (in retail, and on public transport, respectively; Fig. 6d), which happened after the end of the effective phase of the wave 1 lockdown (1 April 2020 [3]). Literature recommendations on this topic were scattered until the metanalysis review [24] of physical distancing, face masks and eye-protection, published on 1 June 2020, concluded that “no intervention, even when properly used, was associated with complete protection from infection.” Victoria did not even introduce compulsory face masks together with the stage 3 lockdown, despite other countries having relied on face masks already during or after the first COVID-19 wave. The fact that a mandatory mask order was introduced by Victoria independently, and at a different time, than all other control measures, is, epidemiologically seen, a fortunate event. Otherwise, either stage 3 or stage 4 lockdown would have initiated the effective phase, and the important, if not dominating effect of wearing face masks would have been obscured.

The fact that the severe lockdowns were ineffective in both Austria and Victoria (Figs. 4 and 5), in contrast to the lighter ones (at least after combined with face masks in Victoria) questions the value of severe lockdowns. The latter are just extensions of the former ones, by reinforcing existing measures and introducing new ones. These two types of lockdowns are not two separate measures. The major step from soft to hard lockdown in Austria was the restriction of movement, and the closure of more businesses and educational facilities. In Victoria it was the night curfew, movement restriction by further shortening distance and duration, closure of businesses and educational facilities. The major differences between Austria and Victoria were: (1) leaving home only for limited reasons was introduced in Victoria (stage 3 lockdown) earlier than in Austria (hard lockdown); and (2) the night curfew was introduced in Austria (soft lockdown) earlier than in Victoria (stage 4). The “secret ingredients”, the light lockdowns of both countries have in common are distancing plus wearing masks; working from home (at least recommended); and limiting public gatherings and events. This analysis informs us of the most powerful measures, but also of most common means of infections (close contact of many people professionally and privately). These common measures of both countries are reflected in the results of Haug et al. [7] among the most significantly effective interventions with an effectiveness score greater than 50%.

The limitation of our study is that the causal connection between control measure and start of the effective phase is construed by circumstantial evidence. The constraints may be defined e.g.: if the effective phase starts

1. before or on the day the control measure is introduced: no causal connection;
2. within one average *SI* after the day the control measure is introduced: unlikely causal connection;
3. between the end of *SI* and the following 5 days: very likely connection;
4. after the end of on *SI* + 5 days: unclear connection, depending on the circumstances, which could be interpreted as a severe delay of the intervention’s effect, or even as an unsuccessful effect, not entirely connected to the intervention, considering that relaxed measures, comparable to the ones introduced in Sweden, also initiated the effective phase.

The transition between these four periods is by no means a sharp one and depends very much on the circumstances, e.g., compliance, penalties, police enforcement, etc. In summary, there is no clear rule other than people, infected within one average SI before a specific control measure is introduced, can still infect other people within one average SI thereafter.

That the two lighter restrictions of Germany (Fig. 6d) already had the desired effect before the “contact ban” was introduced (still lighter than the lockdowns in Austria and Victoria; Figs. 4 and 5) is not further surprising, as even relaxed measures such as appealing to the responsibility of the citizens proved successful. Dehning et al. [21] forecast the individual effect of the three German restrictions by modelling and concluded that the third restriction had the strongest effect. This stands in sharp contrast to our results, which in turn show that the acceleration was already declining when the 3rd restriction was introduced.

## Conclusion

Our study may have substantial implications for decision-makers. While there is no unique recipe that applies to all countries, our method for detecting which preventive control measures initiated the “flattening of the curve”, although not immediately suitable for effectiveness evaluation, will inform retrospectively which control measures were effective or ineffective, a knowledge essential for learning from historic events and preventing disadvantageous decisions in the future. The most important results of our study were the unexpected effectiveness of the mask order in Victoria (decoupled from the stage 3 lockdown); and that the severe lockdown levels (stage 4 in Victoria, and hard lockdown in Austria) were not able to contribute any effectiveness on top of the preceding, less severe lockdowns, so that the preceding, more economy-friendly ones would have sufficed to maintain the dynamics of COVID-19 control.
